# Wernicke encephalopathy with active hallucinations during lung cancer treatment and hemodialysis: A case report

**DOI:** 10.1017/S1478951524001974

**Published:** 2025-01-14

**Authors:** Marino Hirata, Mayumi Ishida, Hideki Onishi

**Affiliations:** 1 Department of Supportive Medicine, Saitama Medical University International Medical Center, Saitama, Japan; 2 Department of Psycho-Oncology, Saitama Medical University International Medical Center, Saitama, Japan

**Keywords:** Wernicke encephalopathy, thiamine deficiency, active hallucinations, hemodialysis, Korsakoff syndrome

## Abstract

**Objectives:**

Wernicke encephalopathy (WE) is an acute neuropsychiatric disorder caused by thiamine deficiency. The classical triad of symptoms for WE include mental status changes, ataxia, and ophthalmoplegia. In contrast, more uncommon symptoms include hallucinations. Known risk factors include alcoholism, malignancies, and chronic kidney disease, particularly hemodialysis. However, WE in nonalcoholic adults is often overlooked.

**Methods:**

We report a WE patient with lung cancer undergoing hemodialysis who presented with the uncommon symptom of active hallucinations, which were improved by thiamine replacement therapy, despite a borderline whole blood thiamine concentration.

**Results:**

An 81-year-old woman with lung cancer and undergoing hemodialysis was referred to our psycho-oncology department for active hallucinations that appeared suddenly 24 days earlier. She had been diagnosed with lung cancer 6 months earlier and was undergoing chemotherapy and radiotherapy. She had no alcohol dependence or anorexia before or after admission. Physical examination revealed active visual hallucinations and delirium. On suspicion of WE, intravenous thiamine was administered. One day after administration, the hallucinations and delirium improved. Her whole blood thiamine concentration was borderline (24 ng/ml).

**Significance of results:**

WE might be a cause of active visual hallucinations as they disappeared on thiamine administration alone. We need to be aware of risk factors such as malignancies and hemodialysis, and it is important not to overlook WE.

## Introduction

Wernicke encephalopathy (WE) is an acute neuropsychiatric disorder caused by thiamine deficiency (TD). Thiamine (vitamin B1), in its biologically active form of thiamine pyrophosphate, is an essential coenzyme for oxidative metabolism (Sechi et al. 2016). Like other essential nutrients, thiamine cannot be produced by the body and must be obtained from the diet. The store of thiamine in the body can be depleted in as little as 18 days, and TD can occur if inadequate nutritional intake lasts for 2–3 weeks (Sechi et al. 2016). The known risk factors for TD are alcoholism, malnutrition, gastrointestinal surgical procedures, loss of thiamine due to recurrent vomiting or malabsorption (Sechi et al. 2016), malignancies (Isenberg-Grzeda et al. 2016; Onishi et al. 2021), and chronic kidney disease, particularly in those undergoing dialysis (Oudman et al. 2024; Seto et al. 2022). However, WE in nonalcoholic adults, particularly cancer patients, is often overlooked (Isenberg-Grzeda et al. 2016).

The classical triad of symptoms for WE includes mental status changes, ataxia, and ophthalmoplegia. However, only 16% of cases present the classical triad, 27.8% of cases show 2 signs, 37.1% of cases show 1 sign, and 19% of cases are asymptomatic. Change in mental status is the most common symptom, present in over 80% of patients, but it varies in degree from mild delirium to global confusion (Harper et al. 1986). Uncommon symptoms or signs include hallucinations, behavioral abnormalities, stupor, epileptic seizures, and hearing loss (Sechi and Serra 2007). The estimated mortality of untreated WE is 20%, with about 80% of surviving patients developing Korsakoff syndrome, a chronic irreversible form of WE (Sechi et al. 2016). Therefore, the provision of thiamine replacement therapy for reversible WE is vital. For diagnosis, in addition to symptoms and clinical course, confirmation of TD based on whole blood thiamine concentration and structural abnormalities around the third ventricle, aqueduct, and fourth ventricle based on head Magnetic Resonance Imaging (MRI)findings are considered useful (Sechi et al. 2016).

Here, we report a WE patient with lung cancer and undergoing hemodialysis who presented with the uncommon symptom of active hallucinations, which were improved by thiamine replacement therapy, despite borderline whole blood thiamine concentration. Active visual hallucinations can disrupt daily life (e.g., aggressive behavior, falls, social withdrawal) (Badcock et al. 2020), placing the patient at risk of discontinuing cancer treatment. There are various causative diseases associated with visual hallucinations, and the resolution of the hallucinations depends on the cause; WE is treatable with thiamine alone, and this case emphasizes the importance of differential diagnosis in such cases.

## Case

An 81-year-old woman with lung cancer was referred to our Psycho-oncology Department for active hallucinations. Six months prior to the first consultation, she was diagnosed with a mass in her right lung and was referred to our Respiratory Medicine Department for a detailed examination. The histological type was Class V small cell carcinoma based on cytology of the bronchial lavage fluid and small cell carcinoma based on a histological examination by transbronchial lung biopsy. After diagnosis, the patient underwent 4 courses of chemotherapy consisting of carboplatin and etoposide as a standard treatment. After chemotherapy, the mass was observed to have shrunk. Thirty-seven days prior to her first consultation, she was admitted to the hospital for radiotherapy. Radiotherapy was scheduled for 60 Gy/30 F irradiation to the right lower lobe. Seven days prior, she experienced fever and radiotherapy was temporarily suspended. Radiotherapy was resumed on the day of consultation, and 20 F had already been completed. Her comorbidities included diabetes, dyslipidemia, hypertension, and chronic renal failure, for which she had been undergoing hemodialysis 3 times a week for about 7 years. She had no alcohol dependence or anorexia before or after admission and was eating a well-balanced diet of about 1600 kcal as usual. At the time of admission, she was 148 cm in height, 48 kg in weight, and had a body mass index of 21.9 kg/m^2^ without any weight loss.

On examination, she complained that, “About 24 days ago, I suddenly started to see people and got scared. Adults and children. When I try to stand up, some people put their hands out toward me, but when I try to hold their hands, they disappear.” She experienced disorientation and disturbance of attention, and her psychiatric features fulfilled the diagnostic criteria for delirium in the Diagnostic and Statistical Manual of Mental Disorders, Fifth Edition, Text Revision (American Psychiatric Association 2022). She had no abnormal vital signs, and no ataxia or ophthalmoplegia was observed. Although she was eating normally and had no weight loss, we suspected WE as TD is commonly observed in cancer patients experiencing delirium (Onishi et al. 2021). After testing her whole blood thiamine concentration, thiamine 100 mg was administered prophylactically. One day after administration, the hallucinations and delirium improved. A few days later, her whole blood thiamine concentration, as measured using ultra-performance liquid chromatography tandem mass spectrometry (Hampel et al. 2012), was found to be 24 ng/ml, which is borderline (reference range: 24–66 ng/mL). Thiamine 100 mg was then administered intravenously for 7 consecutive days. Based on these findings, we diagnosed WE. After administration for 8 days, thiamine 100 mg was switched to oral administration and treatment was continued thereafter. Fourteen days after consultation, the scheduled radiotherapy was completed. Eighteen days after consultation, she was transferred to another hospital for recuperation. She reported that she had no more visions at all for the 18 days before she was transferred.

## Discussion

We reported a WE patient with lung cancer and undergoing hemodialysis who presented with the uncommon symptom of active hallucinations, which was improved by thiamine replacement therapy, despite borderline whole blood thiamine concentration. The following points are worthy of note: the patient presented with active hallucinations as a symptom, her symptoms worsened despite adequate dietary intake, and her whole blood thiamine concentration was borderline.

First, WE is known to result in changes in mental status in over 80% of patients (Harper et al. 1986), while hallucinations, behavioral abnormalities, stupor, epileptic seizures, and hearing loss have been reported as uncommon symptoms or signs (Sechi and Serra 2007). The most common change in mental status is cognitive impairment, but 7% of patients have been reported to experience hallucinations (Harper et al. 1986; Isenberg-Grzeda et al. 2016). Active visual hallucinations can disrupt daily life (e.g., aggressive behavior, falls, social withdrawal). When an elderly person reports hallucinations, particularly visual hallucinations, the differentials include Lewy body dementia, Parkinson’s disease, eye disease, and drug-related conditions (Badcock et al. 2020). Delirium is also a differential, but active delirium with hallucinations and other symptoms often has multiple etiologies (Burns 2004). Hallucinations should be treated based on the cause. For some disorders, such as intoxication and psychotic depression, hallucinations may respond well to treatment of the underlying disorder (Badcock et al. 2020). Among the various causes of visual hallucinations, we should keep WE in mind as a causative disease that can be resolved with thiamine replacement therapy alone. Improvement of visual hallucinations would improve quality of daily life and prevent unwanted interruptions to cancer treatment.

Second, we must consider why the patient developed WE despite adequate dietary intake. Patients with cancer have been reported to be at risk of TD due to decreased thiamine availability, accelerated use of thiamine by rapidly growing tumors or in hypermetabolic states, and impaired thiamine-dependent enzyme function due to cofactor deficiency or inactivation by breakdown products of chemotherapy (Isenberg-Grzeda et al. 2016). Chemotherapy within 2 months, but not radiation therapy, was reported to be a risk factor for TD in cancer patients with delirium (Isenberg-Grzeda et al. 2017). Chronic kidney disease, particularly in those undergoing dialysis, is also reported a risk of TD due to impaired gastrointestinal absorption, chronic diuretic therapy and an increased loss of water-soluble vitamins in the dialysate. In addition, loss of clearance induces an accumulation of an antimetabolite of thiamine called oxythiamine. Oxythiamine is pyrophosphorylated to oxythiamine pyrophosphate, which inhibits transketolase, which catalyzes the pentosephosphate pathway. This causes symptoms of TD even when whole blood thiamine levels are normal (Zhang et al. 2016). In both acute and chronic kidney failure, additional complications such as diabetes mellitus, cancer, sepsis, and infections of other etiology were reported to be relatively common contributive factors to the development of WE (Oudman et al. 2024). In this case, we consider that chemotherapy within 2 months, continuous hemodialysis and diabetes mellitus led to TD, even though the patient received adequate dietary intake. Clinicians should be aware that patients at risk for TD may develop TD even on a normal diet. This will make it easier to recall the disease when they see symptoms that are suspicious for TD. It will also allow for early detection of abnormalities when these patients are eating less.

Next, we examined the patient’s whole blood thiamine concentration and found it to be borderline. High-performance liquid chromatography has been used as a method to measure thiamine pyrophosphate, the principal biologically active form, in whole blood (Davies et al. 2011). However, whole blood thiamine concentration does not necessarily reflect tissue concentrations in the brain (Davies et al. 2011; Isenberg-Grzeda et al. 2016; Ono et al. 2023). How exactly thiamine enters the brain remains unclear, but it is thought to be transported across the blood–brain barrier by a slow, carrier-mediated process (Davies et al. 2011; Ott and Werneke 2020). When WE is suspected, improvement in neurological signs after parenteral thiamine administration is a diagnostic test, and is strongly indicative of TD (Sechi et al. 2016). Based on the above, we recommend that diagnostic treatment with thiamine replacement therapy be attempted if WE is suspected on the basis of clinical symptoms and risk factors, even if the thiamine concentration is not below reference range.

In conclusion, in cases where hallucinations are present in a clinical setting, WE should be suspected, thiamine concentration should be measured, and thiamine replacement therapy should be started as soon as possible.
